# Chemical and Biological Studies of *Achillea setacea Herba* Essential Oil—First Report on Some Antimicrobial and Antipathogenic Features

**DOI:** 10.3390/antibiotics12020371

**Published:** 2023-02-10

**Authors:** Ioana Cristina Marinas, Eliza Oprea, Diana Madalina Gaboreanu, Gratiela Gradisteanu Pircalabioru, Mihaela Buleandra, Eugenia Nagoda, Irinel Adriana Badea, Mariana Carmen Chifiriuc

**Affiliations:** 1 Research Institute of the University of Bucharest-ICUB, 91-95 Spl. Independentei, 050095 Bucharest, Romania; 2 Research and Development Department of S.C. Sanimed International Impex SRL, Șos. București-Giurgiu (DN5), No. 6, 087040 Călugăreni, Romania; 3 Faculty of Biology, Department of Botany and Microbiology, University of Bucharest, 1-3 Portocalilor Way, 060101 Bucharest, Romania; 4 Academy of Romanian Scientists, 3rd Ilfov Street, 051157 Bucharest, Romania; 5 Faculty of Chemistry, Department of Analytical Chemistry, University of Bucharest, 90-92 Panduri Street, 050663 Bucharest, Romania; 6 Garden “D. Brandza”, University of Bucharest, 32 Sos. Cotroceni, 060114 Bucharest, Romania; 7 The Romanian Academy, Biological Sciences Division, Calea Victoriei 125, 010071 Bucharest, Romania

**Keywords:** *Achillea setacea*, essential oil, antimicrobial, antioxidant, antiadherence, NO release, biocompatibility

## Abstract

The essential oil of *Achillea setacea* was isolated by hydrodistillation and characterized by GC-MS. The antioxidant and antimicrobial activity of *Achillea setacea* essential oil was evaluated, as well as its biocompatibility (LDH and MTT methods). DPPH, FRAP, and CUPRAC methods were applied for antioxidant activity evaluation, while qualitative and quantitative assays (inhibition zone diameter, minimum inhibitory concentration, and minimum fungicidal concentration), NO release (by nitrite concentration determination), and microbial adhesion capacity to the inert substrate (the biofilm microtiter method) were used to investigate the antimicrobial potential. A total of 52 compounds were identified by GC-MS in *A. setacea* essential oil, representing 97.43% of the total area. The major constituents were borneol (32.97%), 1,8-cineole (14.94%), camphor (10.13%), artemisia ketone (4.70%), α-terpineol (3.23%), and *γ*-eudesmol (3.23%). With MICs ranging from 0.78 to 30 μg/mL, the *A. setacea* essential oil proved to inhibit the microbial adhesion and induce the NO release. To the best of our knowledge, the present study reports for the first time the antimicrobial activity of *A. setacea* EO against clinically and biotechnologically important microbial strains, such as *Shigella flexneri*, *Listeria ivanovii*, *L. innocua*, *Saccharomyces cerevisiae*, *Candida glabrata*, *Aspergillus niger*, *Rhizopus nigricans*, *Cladosporium cladosporioides*, and *Alternaria alternata*, demonstrating its antimicrobial applications beyond the clinical field.

## 1. Introduction

The genus *Achillea* comprises over 100 species worldwide, which are used as medicinal plants against fever, common cold, digestive complaints, slow-healing wounds and skin inflammations [1]. *Achillea setacea* Waldst. & Kit. is an herbaceous perennial plant, common from the steppe to the beech floor, growing in meadows, rare forests, rocky coasts, and ruderal places. *A. setacea* contains essential oils, flavonoids, alkaloids, glycosides, tannins, resins, organic acids, and vitamins C and K [2,3,4,5].

The chemical composition of the *A. setacea* essential oil (EO) depends on the period and the harvesting area, the analyzed taxa, and the studied vegetative organs. However, Karamenderes [6] has shown that 1,8-cineole (34.30–48.50%) was the major component in the majority of *A. setacea* samples harvested from seven different areas of Turkey. Important differences can also be observed regarding the significant compounds in the EO of *A. setacea* reported in different studies. Thus, eucalyptol (18.5%) and sabinene (10.8%) were reported as major compounds by Unlu [7], *α*-bisabolol oxide by Turkmenoglu [8], nerolidol (20%) and *α*-cubebene (14%) by Rezaei [9], and *β*-eudesmol (29.17%) and debromoallolaurinterol (14.90%) by Yener [10].

To our knowledge, there are no data available on the antioxidant activity of *A. setacea* essential oil. However, it has been reported that *Achillea millefolium* contains in very different percentages terpenoids known for their antioxidant properties (i.e., *α*- and *β*-pinene, camphene, limonene, 1,8-cineole, borneol, sabinene, *α*-phellandrene, *α*-terpinene, *α*-terpinolene, *p*-cymene, *α*-thujone, camphor, terpinen-4-ol, humulene, *α*-terpineol, *β*-caryophyllene, and chamazulene) [11].

There are also very few studies published so far that have focused on the antimicrobial activity of *A. setacea* EO, but the reported results seem very promising. The minimum inhibitory concentration (MIC) of EO varied between 0.28 and 2.25 mg/mL for *Clostridium perfringens*, *Acinetobacter lwoffii*, and *Candida albicans*, and camphor, borneol, terpinene- 4-ol, and eucalyptol have been considered responsible for this antimicrobial activity [7]. The EO extracted from *A. setacea* diluted in culture medium (5% concentration) and containing 1,8-cineole (34.30–48.50%) exhibited antimicrobial activity on *Proteus vulgaris* A, *Salmonella thyphimurium*, and *C. albicans*. In comparison, the EO sample in which camphor was predominant (30.2%) was active only on *C. albicans* [6].

The present study reports for the first time the antimicrobial activity of *A. setacea* EO against a wide variety of bacterial and fungal strains, such as *Shigella flexneri*, *Listeria ivanovii*, *L. innocua*, *Saccharomyces cerevisiae*, *Candida glabrata*, *Aspergillus niger*, *Rhizopus nigricans*, *Cladosporium cladosporioides*, and *Alternaria alternata.* Worldwide, it is considered that *Alternaria alternata* is a fungal allergenic source frequently responsible for the positive results of prick tests (SPT), being often associated with severe forms of bronchial asthma [12], the degree of severity of this pathology being linked to the presence of *Rhizopus nigricans* strains [12]. In addition, it is known that there are respiratory diseases with pulmonary localization produced by *Aspergillus* through direct infection (invasive pulmonary aspergillosis) or hypersensitivity reactions (allergic bronchopulmonary aspergillosis) [13].

Small conidia of *Cladosporium cladosporioides* are an essential cause of respiratory arrest in asthmatic patients and allergic rhinitis due to their easy dispersion in the air [14].

*Listeria ivanovii* and *Listeria innocua* are pathogens reported in ruminants and rarely in humans, especially in immunosuppressed individuals. The first was associated with spontaneous abortion in sheep, goats, and cattle, being identified in food products derived from them, while the second produced infections in various experimental models in mice and zebrafish [15,16].

Another pathogen with food relevance is *Shigella flexneri*, responsible for bacillary dysentery in various regions of the globe, especially in countries with a low socio-economic level. Resistant strains to azithromycin, fluoroquinolones, and generation cephalosporins have been recently reported [17].

Although *S. cerevisiae* was considered safe for humans, in recent years its involvement in a wide range of infections (skin, systemic, and significant organ infections, especially in immunocompromised and catheterized patients) has been established, and it is currently considered an opportunistic fungal pathogen [18]. In addition, *Candida glabrata*, an opportunistic fungal pathogen, can cause urinary tract, vaginal tract, and bloodstream infections, especially in transplant and diabetics patients [19].

Therefore, the aim of this study was to perform a physico-chemical characterization and to determine the antimicrobial and antioxidant activity of *A. setacea* essential oil against bacteria, yeast, and mold strains. A secondary aim was to calculate the selectivity index of this EO, as a key step for selecting the optimal EO concentrations with minimal cytotoxicity. The taxon analyzed in this study is *A. setacea* Waldst. & Kit. (bristly yarrow) from the *Asteraceae* family, with an area that includes continental Eurasia. *A. setacea* was determined based on morphological features (hairy stem, lacinia of the leaves ending with a cartilaginous mucron, involucral leaflets ovate, hairy, fimbriated at the lacerated tip, careened on the back) according to the specialized literature [20,21,22].

This study reports for the first time the inhibitory activity on microbial adhesion capacity and the correlation of this antipathogenic feature with the extracellular NO release induced by *A. setacea* EO, as well as the evaluation of its biocompatibility, by 3-(4,5-dimethylthiazol-2-yl)-2,5-diphenyl-2H-tetrazolium bromide (MTT) and lactate dehydrogenase (LDH) tests.

## 2. Results and Discussion

### 2.1. Physico-Chemical Characterization

The average content of the extracted *A. setacea* EO (10 determinations) was 0.41 ± 0.14%, expressed as mL EO/100 g plant. EOs had a blue color, with a density of 0.8576 ± 0.0609 g/mL.

A total of 52 compounds were identified through GC-MS in *A. setacea* EO, representing 97.43% of the total area. The main identified compounds were borneol (a bicycle monoterpene alcohol), eucalyptol (a bicycle monoterpene ether), and camphor (a bicycle monoterpene ketone), which have the largest relative areas 32.97%, 14.94%, and 10.13%, respectively. In addition, the EO is also rich in artemisia ketone (4.70%), an acyclic monoterpene ketone, *α*-terpineol (3.23%), a monoterpene alcohol, and *γ*-eudesmol (3.23%), a sesquiterpene alcohol.

**Figure 1:**
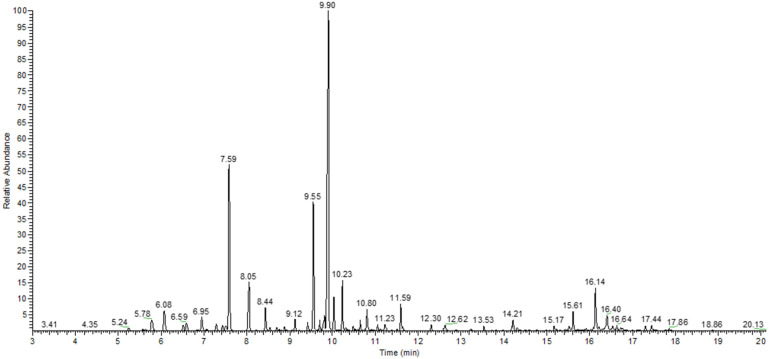
The gas chromatogram of *A. setacea* essential oil.

The EO contains monoterpenes hydrocarbons (7.58%), sesquiterpenes hydrocarbons (1.19%), monoterpenes and sesquiterpenes alcohols (44.24% and 5.42%, respectively), and monoterpenes ketones (15.97%). Table 1 shows the relative content of volatile compounds from the *A. setacea herba* EO growing in Romania, expressed as a percentage of the total area. The gas chromatogram of *A. setacea* EO is shown in Figure 1.

The presence of chamazulene responsible for the blue color of *A. setacea* EO is confirmed only by some of the authors [6,7,23], a possible explanation being the low amounts of this compound produced only during the flowering period, and only by certain taxa, as reported by Karamenderes and Hethelyi [7,23]. In addition, the aerial parts of *A. setacea* could contain some (colorless) compounds that can be considered as proazulenes, such as 11,13-dehydrodeacetylmatricarin (14-deoxylactucin), rupicolin A, and rupicolin B [24], along with 13 other lactones sesquiterpenes identified by Todorova [25].

It is known that azulenogenic sesquiterpene lactones (naturally present in various species of *Achillea*) transform into azulenes during the EO extraction by hydrodistillation but not in case of supercritical extraction. Therefore, the extraction method applied to obtain the EOs could be another source of the compositional differences reported in the literature [26].

The presence of chamazulene in the composition of the EO influences not only the color but also the therapeutic effects, such as photodynamic antimicrobial activity, antiviral, anti-inflammatory, anti-acid effects, etc. [27].

### 2.2. Antioxidant Activity

The radical scavenger activity of the *A. setacea* EO was expressed as the amount of the antioxidants necessary to decrease the initial 2,2-diphenylpicrylhydrazyl (DPPH) absorbance by 50% (median effective concentration value, IC_50_), and results were compared with IC_50_ for butylated hydroxytoluene (BHT) (Table 2). The scavenging activity toward the DPPH radical of the EO of *A. setacea* (IC_50_ = 12.38 ± 0.63 mg/mL) was significantly higher (*p* < 0.0001) than the control, BHT (IC_50_ = 0.64 ± 0.07 mg/mL). The results are substantially different from those published by Rezaei et al. [9], probably due to the differences in the chemical composition, influenced by the pedoclimatic conditions. For this species, the antioxidant activity was determined through ferric reducing antioxidant power (FRAP) and cupric ion reducing antioxidant capacity (CUPRAC) assays for the first time and was significantly lower than that given by BHT (*p* < 0.0001). These methods aim to evaluate the capacity of the sample to reduce ferric or cupric ions in aqueous media. It can be observed that the antioxidant activity for essential oil in an acidic environment (FRAP) is higher than that obtained by CUPRAC, where the environment is neutral. In the case of BHT, the antioxidant activity is higher in neutral pH, suggesting that the antioxidant mechanism is different from that of *A. setacea* EO, probably due to the hydrophobic character [28].

Among the major components of the tested EO, borneol, camphor, and eucalyptol are known for their strong antioxidant activity [29,30,31,32]. Other compounds present in this EO that could contribute to the antioxidant effect are chamazulene [33], eudesmol [34], caryophyllene oxide [35], nerolidol [36], bornyl acetate [37], *α*-pinene [38], camphene [39], terpinen-4-ol [40], etc. According to Karakaya et al., caryophyllene oxide is correlated with a substantial cholinesterase inhibitory activity [35].

### 2.3. Antimicrobial Activity

The qualitative testing of microbial susceptibility to the *A. setacea herba* EO highlighted growth inhibition zones for the following microbial strains: *Escherichia coli*, *Staphylococcus aureus*, *Salmonella typhimurium* vs. *enterica*, *Shigella flexneri*, *Bacillus cereus*, *Listeria ivanovii*, *Listeria innocua*, *Saccharomyces cerevisiae* (SMR4), *Candida glabrata*, and strains isolated from seeds, *Aspergillus niger*, *Rhizopus nigricans*, *Cladosporium cladosporioides*, and *Alternaria alternata* (Table 3). In addition, the solvent (DMSO) in which the essential oil was solubilized showed no antimicrobial activity for any tested strains.

The qualitative results of the antimicrobial activity correlate with those obtained by Unlu et al. [7], the *A. setacea* EO being most active on *B. cereus*, *S. aureus*, and *E. coli*, with higher growth inhibition zones obtained in this study. According to Karamenderes et al. (2003) [6], the *A. setacea* EO also shows activity on *S. thyphimurium*, *P. vulgaris*, and *C. albicans* strains, but it is inactive on the *S. aureus* and *E. coli* strains. These significant differences could be explained by the differences in the composition of the *A. setacea* EO. To our knowledge, the antimicrobial activity of *A. setacea* EO against *Shigella flexneri*, *Listeria ivanovii*, *L. innocua*, *Saccharomyces cerevisiae*, *C. glabrata*, *Aspergillus niger*, *Rhizopus nigricans*, *Cladosporium cladosporioides*, and *Alternaria alternata* are reported for the first time in the literature.

The comparison between the MIC values of the stock solution and the solvent, respectively, revealed statistically significant results for the following strains: *B cereus*, *L. ivanovii*, *E. coli*, and *C. glabrata* with *p* < 0.001; *S. aureus*, *L. inoccua*, *A. niger*, *A. alternata* and *C. cladosporioides* (*p* < 0.01); and *S. enterica* and *R. nigricans* (*p* < 0.05). However, in the case of the *S. cerevisiae* strain, the difference is not statistically significant compared to the solvent control.

In Table 3, the results obtained for MMC are reported, highlighting that the MIC values are clearly lower than the MMCs. In the case of *S. aureus* and *Listeria* sp. the microbial growth was observed at concentrations of 3.75 μL/mL (*S. aureus*), 7.5 μL/mL (*L. inoccua*), and 0.94 μL/mL (*L. ivanovii*). The positive control specific to each strain and the solvent (DMSO) showed confluent colonies after 24 h of incubation in the case of all studied strains, as expected. The obtained MMCs for *A. setacea* EO indicate better activity on Gram-positive bacterial strains and yeasts.

Of particular importance among the components of essential oils with antimicrobial activity are the phenolic derivatives, represented by eugenol in the case of *A. setacea* EO, followed by aldehydes (not present in the studied extract), followed by terpene alcohols (present in large quantities, for example, borneol, terpinen-4-ol, α-terpineol, *cis*-piperitol), esters, and ketones, while monoterpene hydrocarbons used alone present a modest antimicrobial activity [41].

Although a mathematical prediction for complex matrices such as essential oils is difficult to achieve, the correlation data for EO of *Thymus* sp. showed that the synergism generated by the association of p-cymene with *cis*-geraniol (both present in the *A. setacea* EO) could be responsible for the antibacterial activity on *Salmonella typhimurium* and *Shigella flexneri* strains (thymol recorded negative correlations with bacterial strains) [42].

Among the compounds identified in the *A. setacea* EO, terpinen-4-ol and eucalyptol show remarkable antifungal activity against *Fusarium subglutinans*, *F. cerealis*, *F. verticillioides*, *F. proliferatum*, *F. oxysporum*, *F. sporotrichioides*, *Aspergillus tubingensis*, *A. carbonarius*, *Alternaria alternata*, and *Penicillium* sp. [43].

According to Kotan et al. [44], bornyl acetate and eucalyptol do not show antimicrobial activity, while terpinen-4-ol had a broad-spectrum antimicrobial activity against 35 strains including *S. aureus*, *P. aeruginosa*, *E. coli*, and *S. typhimurium*.

Borneol has been shown to exhibit antiadhesion effects by reducing bacterial attachment and biofilm formation [45]. Despite its low antimicrobial activity, borneol, in combination with conventional antibiotics or other antimicrobial compounds, can penetrate the bacterial membrane and impair the efflux pump activity [46], leading to a synergistic interaction and improving the antibacterial activity of other compounds found in low concentrations but with a strong antimicrobial effect, such as terpinen-4-ol [44], limonene [47], pinene [48], carvone [49], and eugenol [50].

Eugenol and its derivatives from the essential oil of *Piper divaricatum* are probably responsible for its antifungal action (obtained by a direct bioautography test after nebulization of fungal spores) on *C. cladosporioides* [51], similar to that of miconazole (MIC 0.5 μg/mL). The essential oils of *Eugenia caryophyllus* and *Cinnamomum zeylanicum* inhibited the *A. alternata* isolate, this activity correlating with the high percentages of eugenol, 90.5% and 80.7%, respectively [51,52]. Although present in a much lower percentage in the case of the essential oil studied in this article, eugenol certainly influences its antimicrobial activity.

Jadhav (2013) [53] showed that the *A. millefolium* essential oil has a bactericidal effect on *L. monocytogenes* and *L. innocua* planktonic cells. *α*-pinene, 1,8-cineole, terpinene-4-ol, and caryophyllene were the main components of this essential oil, the first two being attributed to the respective effect.

A hydroalcoholic extract (ethanol: water = 1:1, V:V) of *A. millefolium* L. inhibited the growth of *A. niger* and *Penicillium hirsutum* by 70.19 and 47.40%, respectively, while for the EO of the same plant, the inhibition ratio was greater than 85% for both strains (for 20 μL/mL of oil) [54], maybe due to the morphological changes induced by EO.

A recent study on EO isolated from two species of *Artemisia* (*Asteraceae* family) shows that sesquiterpenoids, such as bisabolol, are responsible for inhibiting the growth of *A. niger*, *A. flavus*, and *Candida* sp. [55].

*Tanacetum chiliophyllum* var. *chiliophyllum*, which belongs to one of the largest genera in the *Asteraceae* family, contains 1,8-cineole (16.1%), camphor (36.2%), borneol (2.8%), chamazulene (2.9%), and terpinene-4-ol (2.2%) in the essential oil extracted from stems. The antibacterial activity of this essential oil (with a composition close to *A. setacea* EO, at least in terms of the majority of compounds) isolated from plants collected from Van-Muradiye in Istanbul was remarkable because it has had the same MIC as chloramphenicol (62.5 μg/mL) for *E. coli* NRRL B-3008 [56].

Another species from the *Asteraceae* family, *Dittrichia viscosa*, contains an essential oil in which borneol and bornyl acetate represent over 50% and which had antimicrobial activity on *E. coli*, *S. aureus*, *C. albicans*, and *S. cerevisiae* (with MIC between 0.1 and 3.3 mg/mL) [57].

### 2.4. Correlation between Inhibition of Microbial Adherence and the Release of Extracellular NO

Regarding the influence on the adhesion capacity, a significant decrease in the absorbances measured at the wavelength of 490 nm was observed, the graphic representations being expressed as a function of the absorbance for each well specific to the concentrations used from the *A. setacea* essential oil stock solution in DMSO (1:1) in comparison with the used solvent, DMSO, and the microbial growth control.

The ability to adhere to the inert substrate was inhibited for all tested microbial strains at concentrations between MIC/2 and MIC/4 (μL/mL), the tested EO being more active on *Listeria* sp. (Table 4).

NO is used as a dispersal signal in many microbial species, modulating the tolerance to antimicrobial substances. An additional study demonstrated that nitrite-induced stress in *S. aureus* resulted in the impairment of both polysaccharide intracellular adhesion (PIA) synthesis and biofilm formation. Nitrite-induced stress led to the upregulation of genes associated with oxidative and nitrosative stress (including genes for DNA repair, iron homeostasis, and ROS detoxification) [58].

Oxidative and nitrosative stress is produced inside biofilms according to Miranda et al. [59], thus affecting their growth under different conditions and producing ROS and RNI, with a decrease in the extracellular matrix. These radicals could accumulate in the extracellular environment and thus affect the matrix.

Nitrosative stress involves the production and subsequent damage induced by RNIs, which include nitric oxide (NO), peroxynitrite (ONOO^−^), nitric acid (HNO_2_), dinitrogen trioxide (N_2_O_3_), and others. RNIs are small, potentially highly reactive molecules that can be continuously produced in organisms as by-products of anaerobic respiratory metabolism. When the production of ROIs and/or RNIs overwhelms the cell’s ability to eliminate such molecules, they can generate damage to DNA, lipids, and proteins [60]. Due to their hydrophobic nature and lower density compared to water, the EO accumulates on the surface, leading to the occurrence of an anaerobic environment. The lack of oxygen induces cellular stress, and the amount of nitric oxide is increased. Although the NO synthesized endogenously by bacterial NOS inhibits aerobic respiration, the tricarboxylic acid cycle is still active, generating the NADH that could reduce the alternative electron acceptor nitrate, thus maintaining the membrane potential during microaerobiosis [61].

Table 5 shows a significant increase in the extracellular concentration of NO in most tested strains, except *S. cerevisiae* and *A. alternata* (*p* > 0.05) induced by the tested EO at MIC/2 concentration. The MIC/4 concentration does not significantly influence the NO release in the case of *L. ivanovii*, *S. flexneri*, *E. coli*, *S. cerevisiae*, and *A. alternata* strains. The highest extracellular concentration of NO was observed in the case of the *L. inoccua* strain (MIC/2: 4.63 ± 0.43 µM/mL, MIC/4: 5.49 ± 0.43 µM/mL), followed by *C. glabrata* (MIC/2: 3.47 ± 0.14 µM/mL). In the case of fungi, much higher concentrations of NO were observed compared to bacteria, probably due to the hypoxia induced by *A. setacea* EO. The denitrifying system coupled to the mitochondrial electron transport chain facilitates anaerobic respiration associated with ATP synthesis under hypoxic conditions. Nitrite reductases located in the intermembrane space of fungal mitochondria have been shown to reduce NO_2_^−^ to NO in an NADP-dependent manner according to Arasimowicz-Jelonek and Floryszak-Wieczorek [62].

NO release has been significantly correlated with microbial adherence inhibition (*p*-value < 0.05), but the relationship depends on the microbial strains used, being much stronger for the *C. cladosporioides* and *R. nigricans* fungal strains (Table 6).

### 2.5. Biocompatibility of A. setacea Essential Oil

**Figure 2:**
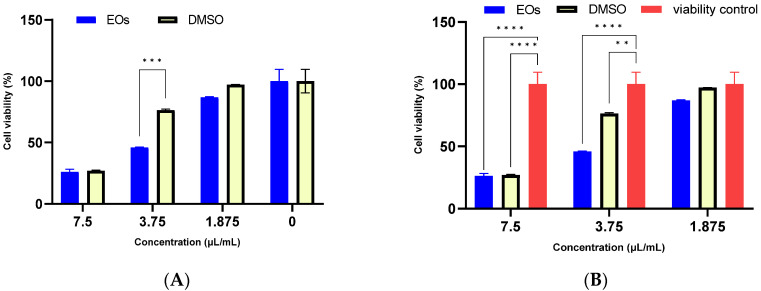
Cytotoxicity assessment of *A. setacea* essential oil by MTT test under standard cultivation conditions. Comparison between the stock solution of essential oil: DMSO (1:1) and DMSO (**A**), and the cell viability control (**B**). The tests were performed in duplicate, and the differences were considered significant for *p* < 0.05 (** *p* < 0.01, *** *p* < 0.001, **** *p* < 0.0001).

The obtained results of the MTT and LDH release assays in cultured mouse fibroblast L929 cells showed that the tested concentrations of *A. setacea* EO stock solution have a cytotoxic effect similar to the solvent used (DMSO) depending on the concentration used. Figure 2 shows the comparison between the solvent and the sample and the control of viable cells. Practically, the cytotoxicity of the EO stock solution is given by an additive effect of the mixture because the used solvent is highly cytotoxic. However, at the concentration of 1.875 µL/mL of essential oil, the cytotoxicity was considerably reduced. The IC_50_ for the essential oil stock solution was 4.68 ± 0.08 µL/mL, while for DMSO it was 5.72 ± 0.04 µL/mL.

**Figure 3:**
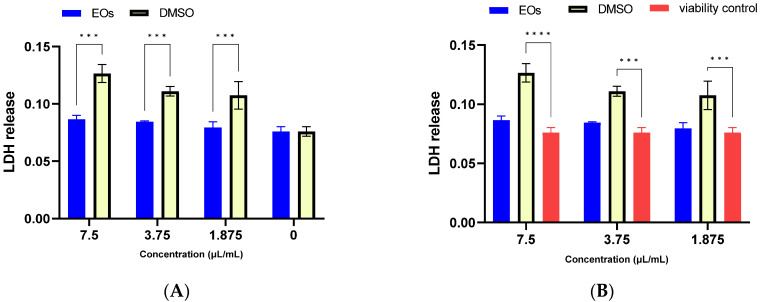
Cytotoxicity assessment of *A. setacea* essential oil by LDH release test under standard cultivation conditions. Comparison between the stock solution of essential oil: DMSO (1:1) and DMSO (**A**), and the cell viability control (**B**). The tests were performed in duplicate, and the differences were considered significant for *p* < 0.05 (*** *p* < 0.001, **** *p* < 0.0001).

From Figure 3, the solvent used increases the release of LDH, while the essential oil solubilized in a similar solvent concentration significantly reduced the release of LDH, which leads to the conclusion that the oil protects the cell from the aggressive action of the solvent. The differences between the essential oil stock solution and the cell viability control are not significant (*p* > 0.05).

Therefore, it can be concluded that the cytotoxic effect is given by the used solvent and not by the *A. setacea* essential oil. Moreover, due to its density being lower than water, the essential oil tends to remain on the surface and thus changes the microenvironment of eukaryotic cells.

By calculating the selectivity index (SI), the MIC obtained values indicate higher toxicity on microorganisms compared to mammalian cells (Table 7). The antifungal effect is much more pronounced than the antimicrobial one in the case of *B. cereus*, *L. inoccua*, *L. ivanovii*, *C. glabrata*, *S. cerevisiae*, *R. nigricans*, *A. alternata*, and *C. cladosporioides* strains, while the used solvent is more toxic to mammalian cells.

## 3. Materials and Methods

### 3.1. Plant Material

The plants were collected from Bucharest, Dămăroia area, near Poligrafiei Boulevard, from a field in the vicinity of the railway in July 2020 (in the blossom period). An herbarium voucher with the number [BUC 410047] was deposited in the herbarium of the “Dimitrie Brandza” Botanical Garden of the University of Bucharest (BUC).

### 3.2. Essential Oil Isolation

Ninety grams of *A. setacea* leaves were hydrodistilled in a Clevenger-type apparatus for 4 h. The essential oil was dried over anhydrous Na_2_SO_4_, stored in a dark glass bottle, and kept at 4 °C until analysis [63].

### 3.3. Gas Chromatography-Mass Spectrometry Analysis

The GC-MS analysis was carried out with a Thermo Electron system (Focus GC chromatograph coupled with a Polaris Q ion trap mass detector) controlled with Xcalibur^®^ software. DB-5MS column (25 m × 0.25 mm; 0.25 μm film thickness) was used with helium as the carrier gas (1 mL/min). Both headspace (500 μL headspace gas) and liquid (1 μL injection from 1:10 hexane dilution) samples were analyzed under the same chromatographic conditions. The GC oven temperature program was kept at 60 °C for 3 min and programmed to 200 °C at a rate of 10 °C/min and after that at 12 °C/min to the final temperature of 240 °C (2 min). The injector temperature was set at 250 °C. Mass spectra were recorded at 70 eV. Mass range was from *m*/*z* 35 to 450.

Identification of the volatile components was carried out by comparison of their relative retention index to series of n-alkanes (C_8_-C_20_ in hexane, Sigma Aldrich Co., St. Louis, MO, USA) and the literature [64].

### 3.4. Antioxidant Activity

#### 3.4.1. DPPH

The method of Ahmed et al. [65], with slight modification, was used to assay the DPPH radical scavenging activity. The stable free radical DPPH was dissolved in methanol to a final concentration of 300 μmol/L solution; 25 μL of the essential oil in methanol (or methanol itself as blank control) was added to 175 μL of the methanol DPPH solution. Different concentrations were tested (100, 50, 25, 12.5, and 6.25 mg/mL for oils in methanol). After further mixing, the decrease in absorbance was measured at 515 nm after 20 min. The antioxidant activity of each test sample was expressed as an IC_50_ value, i.e., the concentration in mg/mL that inhibits DPPH absorption by 50%, and it was calculated from the concentration–effect linear regression curve. 2,6-Di*tert*-butyl-4-methylphenol (IUPAC) or butylated hydroxytoluene (BHT) was used as a positive control. The DPPH radical scavenging activity was calculated as the percentage inhibition.

% Inhibition of DPPH radical activity = [(A_0_ − A_1_)/A_0_] × 100% (1)

where A_0_ is the absorbance of the DPPH with the solvent used; A_1_ is the absorbance of the sample or the positive control (BHT).

#### 3.4.2. FRAP

The determination of the antioxidant capacity of iron reduction was performed by the FRAP assay method [66]. The stock solutions included 300 mM acetate buffer, pH 3.6, 10 mM 2,4,6-tri(2-pyridinyl(-1,3,5-triazine (TPTZ) solution in 40 mM HCl, and 20 mM FeCl_3_ 6H_2_O solution in a volume ratio of 10:1:1, which were warmed to 37 °C before use. A 150 µL aliquot of FRAP reagent was mixed with 25 µL of methanolic essential oil solution (100 mg/mL). After incubation, the absorbance was read at 593 nm. A 1 mM Trolox stock solution was used to plot the calibration curve, the concentration ranging between 30 and 250 µM Trolox/mL (R^2^ = 0.9989). The results were expressed in mM Trolox equivalent/mL (TE) of extract. BHT was used as a positive control.

#### 3.4.3. CUPRAC

Copper ion reduction was performed as previously described [67]: 120 µL of the sample (1 mg/mL in methanol)/standard solutions of different concentrations were mixed with 100 µL CuCl_2_ (10 mM), 100 µL neocuproine (7.5 mM), and 100 µL ammonium acetate buffer 1 M, pH = 7.00. After 30 min, the absorbance was measured at 450 nm. The stock Trolox solutions required for the calibration curve were 2 mM, and the working concentrations were between 0.24 and 2.0 mM Trolox/mL (R^2^ = 0.9980). The results were expressed in mM Trolox equivalent/mL (TE) extract. BHT was used as the positive control.

### 3.5. Antimicrobial Activity

#### 3.5.1. Microbial Strain

The antimicrobial activity was tested on Gram-positive (*Staphylococcus aureus* ATCC 25923, *Bacillus cereus* ATCC 11778, *Listeria inoccua* ATCC 33096, *Listeria ivanovii* ATCC 19110), and Gram-negative (*Escherichia coli* ATCC 25922, *Salmonella typhimurium* vs. *enterica* ATCC 14028, *Shigella flexneri* ATCC 12022) bacterial as well as yeast (*Candida glabrata* ATCC MYA-2950, *Saccharomyces cerevisiae* SMR4) reference strains.

Four species of fungi, *Aspergillus niger*, *Alternaria alternata*, *Rhizopus nigricans*, and *Cladosporium cladosporioides* strains, were obtained from the Microbial Strain Collection of Faculty of Biology, University of Bucharest, Romania, and confirmed by MALDI-TOF.

#### 3.5.2. Qualitative Assay of Antimicrobial Activity

Qualitative assay was performed using microbial suspensions adjusted to 1.5 × 10^8^ CFU/mL according to 0.5 McFarland from 18–24 h cultures. The inoculation in the cloth was carried out on solid media (ALOA for *Listeria* sp and Muller Hilton for the rest of the bacteria, Sabouraud for yeasts, and Dichloran Glycerol Agar (DG18) for fungi). Sterile discs were distributed, on which 5 µL of stock solution was impregnated. The plates were incubated for 24 h at a temperature of 37 °C for bacteria and 72 h at 28 °C for fungi. The zone of inhibition (mm) at the spot level was measured.

#### 3.5.3. Quantitative Assay of Antibacterial Activity

Quantitative antimicrobial activity was carried out by the method of serial binary microdilutions in liquid medium (Demi-Fraser for *Listeria* sp., and TSB (Trypton Soy Broth) for the rest of the bacteria, Sabouraud for yeast) in 96-well plates using a negative control (DMSO). The dilutions of the samples belong to the range of 0.94–30 µL/mL for each strain tested. Simultaneously, serial dilutions were performed with the samples without stems in order to observe the absorbance specific to the sample. Each well was inoculated with 15 µL of 0.5 McFarland microbial suspension. After 20–24 h of incubation at 37 °C, the MIC value was established both macroscopically, as the last concentration at which microbial growth was not observed with respect to the appearance of turbidity of the environment, and spectrophotometrically. The absorbance of the microbial cultures was measured at 620 nm using a FlexStation 3 UV-Vis spectrophotometer (Molecular Devices, GA, USA). The interpretation of the results was achieved by subtracting the absorbances specific to the concentration of each non-inoculated dilution from the inoculated sample.

To determine the minimum microbicidal concentrations (MMC), 10 µL was spotted from the wells where no microbial growth was observed in plates with specific solid media, Aloa for *Listeria* sp., Muller Hilton for the rest of the bacterial strains, and Sabouraud for yeasts.

#### 3.5.4. Quantitative Assay of Antifungal Activity

The fungicidal activity was achieved by the method of binary serial microdilutions in a liquid medium, RPMI 1640 (5.2 g of powder in 500 mL of distilled water, at a pH greater than 7) and inoculated with 0.1 mL of 1 McFarland fungal suspension (made in a solution of 0.01% Tween 80). Essential oil stock solution concentrations ranged from 0.01 to 12.5 µL/mL. Positive control (represented by the untreated strain), solvent control (DMSO), and negative control (sterility control) were used. The tubes were incubated for 3 days at a temperature of 28 °C. On the third day after incubation, the absorbance was read at 550 nm, and they were sown in the spot on the DG18 medium (medium specific for products with a water activity below 0.95) by pipetting 10 μL of the suspension. Finally, the plates were incubated for 3–4 days at a temperature of 28 °C. The minimum fungicidal concentration was represented by the last concentration at which no fungal growth was observed.

#### 3.5.5. Evaluation of NO Release

Nitric oxide (NO) is rapidly converted to nitrite in aqueous solutions, and therefore total nitrite can be used as an indicator of NO concentration. This was measured using a spectrophotometric analysis of total nitrite performed using the Griess reagent according to the methodology described by Quinteros et al. (2016) with some modifications. To the microbial supernatant (MIC/2 and MIC/4) obtained after 24 h incubation for bacteria and 72 h for fungi (50 μL), 50 μL of 2% sulfanilamide in 5% (*v*/*v*) H_3_PO_4_ and 50 μL of 0.1% N-(1-naphthyl)-ethylenediamine aqueous solution were added. The formation of the azo dye was measured after 30 min at λ = 540 nm. For the quantification of nitric oxide, a calibration curve was produced with NaNO_2_ in the range of 0–100 μM (R^2^ = 0.9993).

#### 3.5.6. Microbial Adhesion Capacity to the Inert Substrate

The influence on the adhesion capacity to the inert substrate was quantified after performing the protocol for the quantitative analysis of the antimicrobial effect and establishing the MIC. Using the microtitration method, the biomass adhered to the wells of the microplates was evaluated after washing with AFS, fixation with cold methanol (10 min), and staining with crystal violet (concentration 0.1%, staining time 15 min). The absorbance of the biological material adhered and resuspended in acetic acid (CH_3_COOH 33%, 15 min) was determined by reading the absorbance at 490 nm.

### 3.6. Biocompatibility of Essential Oil

Mouse fibroblast L929 cells were cultured in Gibco Dulbecco’s Modified Eagle Medium (DMEM) supplemented with 10% fetal bovine serum and Penicillin-Streptomycin (100 U/mL) and Amphotericin B (25 µg/mL). *A. setacea* essential oil and DMSO were serially diluted in liquid media. The concentrations tested were 7.5, 3.75, and 1.875 µL/mL.

#### 3.6.1. LDH

Cytotoxicity was assessed using the LDH Released Detection Kit (Roche). LDH activity was measured spectrophotometrically at λ = 490 nm with a wavelength reference λ = 600 nm.

#### 3.6.2. MTT

The MTT assay was used to assess cell viability and proliferation in the presence of *A. setacea* essential oil stock solution and DMSO. The Vybrant^®^ MTT Cell Proliferation Assay Kit (cat no. V-13154) was used for the determination according to the manufacturer’s instructions.

#### 3.6.3. Selectivity Index

The IC_50_ (cytotoxicity) values were calculated as the concentration of essential oil, resulting in a 50% reduction in absorbance compared to untreated cells [68]. The SI was defined as IC_50_ of essential oil against eukaryotic cell line/MIC of essential oil for every microbial strain. SI > 1 means that the extract is more toxic to bacteria/fungi than to mammalian cells [69]. Selective activities of the essential oil were calculated as follows:

(2) $$\mathrm{SI}\;\left(\%\right)=\frac{{\mathrm{IC}}_{50}\;\left(µL/\mathrm{mL}\right)}{\mathrm{MIC}\;\left(µL/\mathrm{mL}\right)}$$

### 3.7. Statistical Analysis

Data obtained in triplicate/duplicate were expressed as means ± SD. Statistical analysis was performed using GraphPad Prism v9. Data were analyzed using the unpaired t-test for antioxidant activity. The correction of multiple comparisons was carried out by the Holm–Sidak method, the comparison being sample vs. solvent control or sample vs. cell viability control. The correlation between microbial adherence and the ability of microorganisms to release extracellular NO was achieved through the Pearson correlation. The cytotoxicity assays were analyzed by ordinary two-way Anova using a two-stage linear step-up procedure of Benjamini, Krieger, and Yekutieli, with a single pooled variance method. The level of statistical significance was set at *p* < 0.05.

## 4. Conclusions

The *Achillea setacea* essential oil proved to be rich in borneol, eucalyptol, and camphor compounds, well known for their antimicrobial and antioxidant activity. The obtained EO has shown a strong antifungal effect and inhibited microbial adhesion, especially in the case of Gram-positive bacteria (with the *S. aureus* strain exception) and *C. glabrata* strains. The NO release favors the inhibition of microbial adhesion to the inert substrate in a strain-dependent manner. The selectivity index highlighted that the maximum concentration of EO that does not influence the mammalian cells is 3.75 µL/mL. Therefore, this EO can be used as an antibacterial and antifungal agent for food product decontamination, and in topical formulations for fungal skin infections.

## Figures and Tables

**Table 1:** The chemical composition of *A. setacea* EO resulted from gas chromatography coupled with mass spectrometry.

No.	Compound Name	Compound Classes	RI ^[l]^	Relative Area (%)
1	Santolina triene	MH ^[a]^	915	0.35
2	Tricyclene	MH ^[a]^	928	0.15
3	*α*-Thujene	MH ^[a]^	932	0.17
4	*α*-Pinene	MH ^[a]^	940	1.40
5	Camphene	MH ^[a]^	956	2.45
6	Sabinene (*β*-Thujene)	MH ^[a]^	983	0.58
7	*β*-Pinene	MH ^[a]^	985	0.89
8	2,3-Dehydro-1,8-cineole	ME ^[c]^	998	0.19
9	Yomogi alcohol	MA ^[b]^	1006	1.31
10	*α*-Phellandrene	MH ^[a]^	1013	0.17
11	*α* -Terpinene	MH ^[a]^	1025	0.69
12	*p*-Cymene	MH ^[a]^	1033	0.49
13	Limonene	MH ^[a]^	1038	0.46
14	Eucalyptol (1,8-Cineole)	ME ^[c]^	1042	**14.94**
15	Artemisia ketone	MK ^[e]^	1067	4.70
16	*cis*-Sabinene hydrate	MA ^[b]^	1078	0.07
17	Artemisia alcohol	MA ^[b]^	1094	1.73
18	*α*-Terpinolene	MH ^[b]^	1097	0.28
19	*trans*-Sabinene hydrate	MA ^[b]^	1111	0.09
20	*α*-Thujone	MK ^[e]^	1117	0.30
21	*cis-p*-Menth-2-en-1-ol	MA ^[b]^	1132	0.85
22	*trans-p*-Menth-2-en-1-ol	MA ^[b]^	1151	0.71
23	Camphor	MK ^[e]^	1160	**10.13**
24	*cis*-Chrysanthenol	MA ^[c]^	1169	0.76
25	Lavandulol	MA ^[b]^	1177	0.45
26	Borneol	MA ^[b]^	1181	**32.97**
27	Terpinen-4-ol	MA ^[b]^	1191	2.36
28	*α*-Terpineol	MA ^[b]^	1204	3.23
29	*cis*-Piperitol	MA ^[b]^	1220	0.15
30	*cis*-Carveol	MA ^[b]^	1232	0.64
31	*trans*-Crysanthenyl acetate	MAE ^[d]^	1242	1.62
32	Carvone	MK ^[e]^	1259	0.38
33	Geraniol	MA ^[b]^	1264	0.14
34	Piperitone	MK ^[e]^	1271	0.46
35	*cis*-Chrysanthenyl acetate	MAE ^[d]^	1273	0.24
36	Bornyl acetate	MAE ^[d]^	1296	1.49
37	*trans*-Carvyl acetate	MAE ^[d]^	1348	0.43
38	Eugenol	PM ^[f]^	1372	0.29
39	*cis*-Jasmone	CK ^[g]^	1417	0.16
40	*β*-caryophyllene	SH ^[h]^	1441	0.36
41	*α*-Humulene	SH ^[h]^	1476	0.10
42	*γ*-Muurolene	SH ^[h]^	1494	0.25
43	Germacrene D	SH ^[h]^	1503	0.26
44	Viridiflorene (Ledene)	SH ^[h]^	1509	0.11
45	*δ*-Cadinene	SH ^[h]^	1542	0.12
46	*trans-β*-Nerolidol	SA ^[i]^	1575	0.28
47	Spathulenol	SA ^[i]^	1604	0.41
48	Caryophyllene oxide	SE ^[j]^	1612	1.33
49	*γ*-Eudesmol	SA ^[i]^	1659	3.23
50	*α*-Eudesmol	SA ^[i]^	1682	1.46
51	*α*-Bisabolol	SA ^[i]^	1704	0.31
52	Chamazulene	AH ^[k]^	1756	0.35
	**Total**			**97.43**
	Monoterpenes hydrocarbons			8.07
	Monoterpenes alcohols and esters			49.23
	Monoterpenes ketones			15.97
	Sesquiterpenes hydrocarbons			1.19
	Sesquiterpenes alcohols and ethers			6.75

^[a]^ MH, monoterpene hydrocarbon; ^[b]^ MA, monoterpene alcohol; ^[c]^ ME, monoterpene ether; ^[d]^ MAE, monoterpene alcohol ester; ^[e]^ MK, monoterpene ketone; ^[f]^ PH, phenolic monoterpene; ^[g]^ CK, cyclic ketone; ^[h]^ SH, sesquiterpene hydrocarbon; ^[i]^ SA, sesquiterpene alcohol; ^[j]^ SE, sesquiterpene ether; ^[k]^ AH, aromatic hydrocarbon; ^[l]^ RI, Kovats Index, the retention index relative to C_8_-C_20_ n-alkanes (DB-5MS column).

**Table 2:** The antioxidant properties of the *A. setacea* essential oil.

	DPPH (IC_50_, mg/mL)	FRAP (µM TE/mg)	CUPRAC (µM TE/mg)
*A. setacea* essential oil	12.38 ± 0.63	14.70 ± 0.99	6.17 ± 0.68
BHT	0.64 ± 0.07	823.46 ± 14.69	988.03 ± 11.35
*p*-value	<0.0001	<0.0001	<0.0001

TE, Trolox equivalent; DPPH, 2,2-diphenylpicrylhydrazyl; FRAP, ferric reducing antioxidant power; CUPRAC, cupric ion reducing antioxidant capacity; BHT, butylated hydroxytoluene.

**Table 3:** The minimum inhibitory (MIC) and microbicidal (MMC) concentrations for the essential oils stock solution and DMSO on the microbial strains involved in the contamination of food products and the etiology of food poisoning.

	IDZ * (mm)		MIC (µL/mL)				MMC (µL/mL)	
	*A. setacea* EO	DMSO	*A. setacea* EO		DMSO	*p*-Value	*A. setacea* EO	DMSO
			MIC **	MV% ^#^	MV% ^#^			
*S. aureus*	13.00 ± 1.00	-	7.50	1.62 ± 0.28	55.45 ± 2.53	0.001116	7.50	>30.00
*B. cereus*	20.00 ± 2.00	-	3.75	0.71 ± 0.29	82.57 ± 2.76	0.000574	3.75	>30.00
*L. inoccua*	NA ^##^	-	3.75	3.57 ± 0.33	80.46 ± 3.95	0.001326	15.00	>30.00
*L. ivanovii*	NA	-	1.88	0.59 ± 0.42	102.04 ± 2.28	0.000261	1.88	>30.00
*E. coli*	8.33 ± 0.58	-	7.50	23.97 ± 0.76	88.59 ± 2.53	0.000835	7.50	>30.00
*S. flexneris*	8.00 ± 2.00	-	30.00	>30	12.55 ± 8.46	-	30.00	>30.00
*S. enterica*	6.33 ± 0.58	-	15.00	36.75 ± 2.03	49.23 ± 1.45	0.019399	30.00	>30.00
*C. glabrata*	10.00 ± 1.00	-	3.75	4.40 ± 0.93	80.57 ± 1.87	0.000376	3.75	>30.00
*S. cerevisiae*	13.33 ± 0.58	-	1.88	6.19 ± 0.34	46.00 ± 7.74	0.350835	3.75	>30.00
*A. niger*	8.00 ± 1.00	-	0.78	7.32 ± 3.45	82.93 ± 0.00	0.001039	12.50	>12.50
*R. nigricans*	6.33 ± 0.58	-	1.56	32.89 ± 1.86	68.42 ± 7.44	0.022511	>12.50	>12.50
*A. alternata*	7.66 ± 0.58	-	1.56	0.00 ± 0.40	114.61 ± 7.15	0.001946	>12.50	>12.50
*C. cladosporioides*	9.33 ± 0.16	-	3.125	7.69 ± 4.35	70.77 ± 1.09	0.002517	>12.50	>12.50

* IDZ—inhibition diameter zone; ** MIC—minimum inhibition concentration; ^#^ MV—microbial viability; ^##^ NA—Not Active.

**Table 4:** Microbial adherence (%) in the case of MIC/2 and MIC/4 for the essential oil stock solution and DMSO on the microbial strains involved in the contamination of food products.

	EO		DMSO	*p*-Value	EO		DMSO	*p*-Value
	MIC/2	MA (%) *	MA (%) *		MIC/4	MA (%) *	MA (%) *	
*S. aureus*	3.75	34.38 ± 12.64	60.84 ± 2.30	0.100432	1.88	78.57 ± 11.78	65.06 ± 2.27	0.252256
*B. cereus*	1.88	14.19 ± 1.76	68.73 ± 3.71	0.002822	0.94	12.60 ± 2.43	39.93 ± 3.14	0.010389
*L. inoccua*	1.88	1.87 ± 0.64	54.57 ± 0.83	0.000198	0.94	1.92 ± 0.70	58.70 ± 2.33	0.000917
*L. ivanovii*	0.94	7.08 ± 0.24	78.80 ± 2.16	0.000459	<0.94	-	-	-
*E. coli*	3.75	101.76 ± 2.15	134.88 ± 14.75	0.088098	1.88	104.18 ± 5.69	150.39 ± 21.55	0.099298
*S. enterica*	7.50	13.02 ± 3.66	25.99 ± 2.02	0.048218	3.75	11.52 ± 0.27	27.20 ± 3.73	0.027284
*C. glabrata*	1.88	0.18 ± 0.25	70.22 ± 2.72	0.000760	0.94	22.64 ± 0.97	127.84 ± 2.02	0.000227
*C. cladosporioides*	1.56	32.81 ± 4.42	105.88 ± 13.87	0.687280	0.78	40.63 ± 2.21	86.27 ± 11.09	0.029350
*R. nigricans*	0.78	23.64 ± 1.29	58.18 ± 1.29	0.001392	0.39	25.45 ± 0.00	49.09 ± 2.57	0.005858
*A. niger*	0.39	87.5 ± 13.26	229.69 ± 15.47	0.010111	0.20	120.21 ± 2.21	223.42 ± 12.31	0.007262
*A. alternata*	0.78	118.39 ± 13.00	133.33 ± 17.88	0.440060	0.39	145.98 ± 16.26	112.64 ± 1.63	0.102062

* MA—microbial adherence.

**Table 5:** The concentrations of NO released in the extracellular environment for the MIC/2 and MIC/4 values in the presence of EO stock solution and DMSO on the microbial strains involved in the contamination of food products.

	EO		DMSO	*p*-Value	EO		DMSO	*p*-Value
	MIC/2	ENOC *	ENOC *		MIC/4	ENOC *	ENOC *	
*S. aureus*	3.75	1.82 ± 0.14	0.39 ± 0.14	<0.000001	1.88	0.18 ± 0.14	1.41 ± 0.14	<0.00001
*L. inoccua*	1.88	4.63 ± 0.43	3.55 ± 0.58	<0.0001	0.94	5.49 ± 0.43	3.25 ± 0.14	<0.000001
*L. ivanovii*	0.94	2.14 ± 0.14	NA **	<0.000001	0.47	0.92 ± 0.14	0.51 ± 0.14	>0.05
*S. flexneri*	15.00	NA	0.82 ± 0.14	<0.001	7.50	0.82 ± 0.14	0.41 ± 0.14	>0.05
*E. coli*	3.75	2.43 ± 0.14	1.20 ± 0.43	<0.00001	1.88	1.10 ± 0.29	1.00 ± 0.14	>0.05
*S. enterica*	7.50	1.10 ± 0.00	0.59 ± 0.14	<0.05	3.75	1.41 ± 0.14	0.69 ± 0.00	<0.01
*C. glabrata*	1.88	3.47 ± 0.14	0.41 ± 0.14	<0.000001	0.94	0.41 ± 0.14	0.10 ± 0.00	<0.001
*S. cerevisiae*	0.94	0.41 ± 0.00	NA	>0.05	0.47	0.20 ± 0.00	0.31 ± 0.14	>0.05
*C. cladosporioides*	1.56	2.68 ± 0.07	1.92 ± 0.14	<0.01	0.78	2.68 ± 0.07	2.07 ± 0.21	<0.01
*R. nigricans*	0.78	2.73 ± 0.07	1.97 ± 0.14	<0.01	0.39	2.78 ± 0.14	2.07 ± 0.29	<0.05
*A. niger*	0.39	3.64 ± 0.14	2.32 ± 0.14	<0.001	0.20	2.07 ± 0.07	0.61 ± 0.14	<0.0001
*A. alternata*	0.78	2.89 ± 0.64	2.02 ± 0.64	>0.05	0.39	1.87 ± 0.36	2.12 ± 0.07	>0.05

* ENOC—extracellular nitric oxide concentration (µM/mL); ** NA—Not Active.

**Table 6:** The correlation coefficients (Pearson correlation) between NO release and inhibition of microbial adherence capacity.

Strains	R^2^	*p*-Value	Significance (α = 0.05)
*S. aureus*	0.6246	0.0228	Yes
*L. inoccua*	0.8790	0.0437	Yes
*L. ivanovii*	0.6036	0.0578	No
*E. coli*	0.3825	0.2079	No
*S. enterica*	0.9022	0.0152	Yes
*C. glabrata*	0.4970	0.1183	No
*C. cladosporioides*	0.9861	0.0041	Yes
*R. nigricans*	0.9772	0.0060	Yes
*A. niger*	0.5044	0.0677	No
*A. alternata*	0.3558	0.0561	No

**Table 7:** Selectivity index values of *A. setacea* essential oil and DMSO against microbial strains.

	SI (IC_50_/MIC)					
	*A. setacea* EO			DMSO		
	MIC	SI	Interpretation	MIC	SI	Interpretation
*S. aureus*	7.50	0.624	*MTEC	>60.00	-	*MTEC
*B. cereus*	3.75	1.248	**MTMC	30.00	0.191	*MTEC
*L. inoccua*	3.75	1.248	**MTMC	>60.00	-	*MTEC
*L. ivanovii*	1.88	2.489	**MTMC	60.00	0.095	*MTEC
*E. coli*	7.50	0.624	*MTEC	>60.00	-	*MTEC
*S. flexneris*	30.00	0.156	*MTEC	30.00	0.191	*MTEC
*S. enterica*	15.00	0.312	*MTEC	60.00	0.095	*MTEC
*C. glabrata*	3.75	1.248	**MTMC	30.00	0.191	*MTEC
*S. cerevisiae*	1.88	2.489	**MTMC	15.00	0.381	*MTEC
*A. niger*	0.78	6.000	**MTMC	6.25	0.915	*MTEC
*R. nigricans*	1.56	3.000	**MTMC	12.50	0.458	*MTEC
*A. alternata*	1.56	3.000	**MTMC	>25.00	-	*MTEC
*C. cladosporioides*	3.125	1.498	**MTMC	6.25	0.915	*MTEC

*MTEC—more toxic to eukaryotic cell; **MTMC—more toxic to microbial cell.

## Data Availability

Not applicable.

## References

[1] Si X.-T., Zhang M.-L., Shi Q.-W., Kiyota H. (2006). Chemical Constituents of the Plants in the GenusAchillea. Chem. Biodivers..

[2] Marchart E., Kopp B. (2003). Capillary Electrophoretic Separation and Quantification of Flavone-O- and C-Glycosides in *Achillea setacea* W. et K. J. Chromatogr. B.

[3] Eisenman S.W., Zaurov D.E., Struwe L., Eisenman S.W., Zaurov D.E., Struwe L. (2013). Medicinal Plants of Central Asia: Uzbekistan and Kyrgyzstan.

[4] Mohammadhosseini M., Sarker S.D., Akbarzadeh A. (2017). Chemical Composition of the Essential Oils and Extracts of Achillea Species and Their Biological Activities: A Review. J. Ethnopharmacol..

[5] Başer K.H.C., Hüsnü K., Başer C. (2016). Essential Oils of Achillea Species of Turkey. Volatiles Essent. Oils.

[6] Karamenderes C., Karabay N.Ü., Zeybek U. (2003). Türkiye’nin Farkli Lokalitelerinden Toplanan *Achillea setacea* Waldst. Kit. Uçucu Yağinin Bileşimi ve Antimikrobiyal Aktivitesi. Ankara Univ. Eczac. Fak. Derg..

[7] Ünlü M., Daferera D., Dönmez E., Polissiou M., Tepe B., Sökmen A. (2002). Compositions and the in Vitro Antimicrobial Activities of the Essential Oils of *Achillea setacea* and Achillea Teretifolia (Compositae). J. Ethnopharmacol..

[8] Turkmenoglu F.P., Agar O.T., Akaydin G., Hayran M., Demirci B. (2015). Characterization of Volatile Compounds of Eleven Achillea Species from Turkey and Biological Activities of Essential Oil and Methanol Extract of A. Hamzaoglui Arabaci & Budak. Molecules.

[9] Rezaei F., Jamei R., Heidari R., Maleki R. (2017). Chemical Composition and Antioxidant Activity of Oil from Wild *Achillea setacea* and A. Vermicularis. Int. J. Food Prop..

[10] Yener I., Yilmaz M.A., Olmez O.T., Akdeniz M., Tekin F., Hasimi N., Alkan M.H., Ozturk M., Ertas A. (2020). A Detailed Biological and Chemical Investigation of Sixteen Achillea Species’ Essential Oils via Chemometric Approach. Chem. Biodivers..

[11] Buleandra M., Moldovan Z., Badea I.A., David I.G., Popa D.E., Oprea E., Caglar T.A.T., Basaga S.H. (2020). Comparative Assessment of the Volatile Profile, Antioxidant Capacity and Cytotoxic Potential of Different Preparation of Millefolli Herba Samples. Rev. Chim..

[12] Sánchez P., Vélez-del-Burgo A., Suñén E., Martínez J., Postigo I. (2022). Fungal Allergen and Mold Allergy Diagnosis: Role and Relevance of Alternaria Alternata Alt a 1 Protein Family. J. Fungi.

[13] Alen Coutinho I., Lopes M., Lima F., Ventura C., Rabadão E., Alfaro T., da Cunha J.S., Regateiro F.S. (2022). Concomitant Allergic Bronchopulmonary Aspergillosis and Eosinophilic Granulomatosis with Polyangiitis after Aspergillus Niger Infection. Pulmonology.

[14] Sandoval-Denis M., Gené J., Sutton D.A., Wiederhold N.P., Cano-Lira J.F., Guarro J. (2016). New Species of Cladosporium Associated with Human and Animal Infections. Persoonia-Mol. Phylogeny Evol. Fungi.

[15] Lianou D.T., Skoulakis A., Michael C.K., Katsarou E.I., Chatzopoulos D.C., Solomakos N., Tsilipounidaki K., Florou Z., Cripps P.J., Katsafadou A.I. (2022). Isolation of Listeria Ivanovii from Bulk-Tank Milk of Sheep and Goat Farms—From Clinical Work to Bioinformatics Studies: Prevalence, Association with Milk Quality, Antibiotic Susceptibility, Predictors, Whole Genome Sequence and Phylogenetic Relationships. Biology.

[16] Matto C., D’Alessandro B., Mota M.I., Braga V., Buschiazzo A., Gianneechini E., Varela G., Rivero R. (2022). Listeria Innocua Isolated from Diseased Ruminants Harbour Minor Virulence Genes of L. Monocytogenes. Vet. Med. Sci..

[17] Nisa I., Qasim M., Yasin N., Ullah R., Ali A. (2020). Shigella Flexneri: An Emerging Pathogen. Folia Microbiol..

[18] Pérez-Torrado R., Querol A. (2016). Opportunistic Strains of Saccharomyces Cerevisiae: A Potential Risk Sold in Food Products. Front. Microbiol..

[19] Kumar K., Askari F., Sahu M., Kaur R. (2019). Candida Glabrata: A Lot More Than Meets the Eye. Microorganisms.

[20] Prodan I., Săvulescu T. (1964). Achillea. Flora României.

[21] Ciocârlan V. (2009). Flora Ilustrată a României: Pteridophyta et Spermatophyta.

[22] Sârbu I., Ștefan N., Oprea A. (2013). Plante Vasculare Din România–Determinator Ilustrat de Teren.

[23] Héthelyi É., Dános B., Tétényi P., Héthelyi, Dános B., Tétényi P. (1989). Phytochemical Studies on the Essential Oils of Species Belonging to TheAchillea Genus by Gas Chromatography/Mass Spectrometry. Biol. Mass Spectrom..

[24] Zitterl-Eglseer K., Jurenitsch J., Korhammer S., Haslinger E., Sosa S., Loggia R., Kubelka W., Franz C. (1991). Entzündungshemmende Sesquiterpenlactone von *Achillea setacea*. Planta Med..

[25] Todorova M., Vogler B., Tsankova E. (2000). Terpenoids from *Achillea setacea*. Z. Naturforsch. C.

[26] Lemberkovics É., Kéry Á., Kakasy A., Szoke É., Simándi B. (2004). Effect of Extraction Methods on the Composition of Essential Oils. Acta Hortic..

[27] Muhammad S., Abdul Khalil H.P.S., Abd Hamid S., Danish M., Marwan M., Yunardi Y., Abdullah C.K., Faisal M., Yahya E.B. (2022). Characterization of Bioactive Compounds from Patchouli Extracted via Supercritical Carbon Dioxide (SC-CO2) Extraction. Molecules.

[28] Apak R., Özyürek M., Güçlü K., Çapanoğlu E. (2016). Antioxidant Activity/Capacity Measurement. 1. Classification, Physicochemical Principles, Mechanisms, and Electron Transfer (ET)-Based Assays. J. Agric. Food Chem..

[29] Kordali S., Cakir A., Mavi A., Kilic H., Yildirim A. (2005). Screening of Chemical Composition and Antifungal and Antioxidant Activities of the Essential Oils from Three Turkish Artemisia Species. J. Agric. Food Chem..

[30] Chrysargyris A., Mikallou M., Petropoulos S., Tzortzakis N. (2020). Profiling of Essential Oils Components and Polyphenols for Their Antioxidant Activity of Medicinal and Aromatic Plants Grown in Different Environmental Conditions. Agronomy.

[31] Xu G., Guo J., Sun C. (2021). Eucalyptol Ameliorates Early Brain Injury after Subarachnoid Haemorrhage via Antioxidant and Anti-Inflammatory Effects in a Rat Model. Pharm. Biol..

[32] Drikvandi P., Bahramikia S., Alirezaei M. (2020). Modulation of the Antioxidant Defense System in Liver, Kidney, and Pancreas Tissues of Alloxan-induced Diabetic Rats by Camphor. J. Food Biochem..

[33] Capuzzo A., Occhipinti A., Maffei M.E. (2014). Antioxidant and Radical Scavenging Activities of Chamazulene. Nat. Prod. Res..

[34] Aati H., El-Gamal A., Kayser O. (2019). Chemical Composition and Biological Activity of the Essential Oil from the Root of Jatropha Pelargoniifolia Courb. Native to Saudi Arabia. Saudi Pharm. J..

[35] Karakaya S., Yilmaz S.V., Özdemir Ö., Koca M., Pınar N.M., Demirci B., Yıldırım K., Sytar O., Turkez H., Baser K.H.C. (2020). A Caryophyllene Oxide and Other Potential Anticholinesterase and Anticancer Agent in *Salvia verticillata* Subsp. Amasiaca (Freyn & Bornm.) Bornm. (Lamiaceae). J. Essent. Oil Res..

[36] Nogueira Neto J.D., de Almeida A.A.C., da Silva Oliveira J., dos Santos P.S., de Sousa D.P., de Freitas R.M. (2013). Antioxidant Effects of Nerolidol in Mice Hippocampus After Open Field Test. Neurochem. Res..

[37] Kim S.H., Lee S.Y., Hong C.Y., Gwak K.S., Park M.J., Smith D., Choi I.G. (2013). Whitening and Antioxidant Activities of Bornyl Acetate and Nezukol Fractionated from Cryptomeria Japonica Essential Oil. Int. J. Cosmet. Sci..

[38] Aydin E., Türkez H., Geyikoğlu F. (2013). Antioxidative, Anticancer and Genotoxic Properties of α-Pinene on N2a Neuroblastoma Cells. Biologia.

[39] Quintans-Júnior L., Moreira J.C.F., Pasquali M.A.B., Rabie S.M.S., Pires A.S., Schröder R., Rabelo T.K., Santos J.P.A., Lima P.S.S., Cavalcanti S.C.H. (2013). Antinociceptive Activity and Redox Profile of the Monoterpenes (+)-Camphene, p -Cymene, and Geranyl Acetate in Experimental Models. ISRN Toxicol..

[40] Lee K.-G., Shibamoto T. (2001). Antioxidant Activities of Volatile Components Isolated FromEucalyptus Species. J. Sci. Food Agric..

[41] Yammine J., Chihib N.-E., Gharsallaoui A., Dumas E., Ismail A., Karam L. (2022). Essential Oils and Their Active Components Applied as: Free, Encapsulated and in Hurdle Technology to Fight Microbial Contaminations. A Review. Heliyon.

[42] Beicu R., Alexa E., Obiștioiu D., Cocan I., Imbrea F., Pop G., Circioban D., Moisa C., Lupitu A., Copolovici L. (2021). Antimicrobial Potential and Phytochemical Profile of Wild and Cultivated Populations of Thyme (*Thymus* Sp.) Growing in Western Romania. Plants.

[43] Morcia C., Malnati M., Terzi V. (2011). In Vitro Antifungal Activity of Terpinen-4-Ol, Eugenol, Carvone, 1,8-Cineole (Eucalyptol) and Thymol against Mycotoxigenic Plant Pathogens. Food Addit. Contam. Part A.

[44] Kotan R., Kordali S., Cakir A. (2007). Screening of Antibacterial Activities of Twenty-One Oxygenated Monoterpenes. Z. Naturforsch. C.

[45] Luo L., Li G., Luan D., Yuan Q., Wei Y., Wang X. (2014). Antibacterial Adhesion of Borneol-Based Polymer via Surface Chiral Stereochemistry. ACS Appl. Mater. Interfaces.

[46] Fadli M., Bolla J.-M., Mezrioui N.-E., Pagès J.-M., Hassani L. (2014). First Evidence of Antibacterial and Synergistic Effects of Thymus Riatarum Essential Oil with Conventional Antibiotics. Ind. Crop. Prod..

[47] Haiyan L., Chongxin X., Xiao Z., Ying L., Xianjin L. (2016). Antibacterial Effect of Limonene on Food-Borne Pathogens. J. Zhejiang Univ. (Agric. Life Sci.).

[48] Leite A.M., de Lima E.O., de Souza E.L., de Diniz M.F.F.M., Trajano V.N., deMedeiros I.A. (2007). Inhibitory Effect of Beta-Pinene, Alpha-Pinene and Eugenol on the Growth of Potential Infectious Endocarditis Causing Gram-Positive Bacteria. Rev. Bras. Ciênc. Farm..

[49] Moro I.J., Gondo G.D.G.A., Pierri E.G., Pietro R.C.L.R., Soares C.P., de Sousa D.P., dos Santos A.G. (2018). Evaluation of Antimicrobial, Cytotoxic and Chemopreventive Activities of Carvone and Its Derivatives. Braz. J. Pharm. Sci..

[50] Catherine A.A., Deepika H., Negi P.S. (2012). Antibacterial Activity of Eugenol and Peppermint Oil in Model Food Systems. J. Essent. Oil Res..

[51] da Silva J.K.R., Andrade E.H.A., Guimarães E.F., Maia J.G.S. (2010). Essential Oil Composition, Antioxidant Capacity and Antifungal Activity of Piper Divaricatum. Nat. Prod. Commun..

[52] Castro J.C., Endo E.H., de Souza M.R., Zanqueta E.B., Polonio J.C., Pamphile J.A., Ueda-Nakamura T., Nakamura C.V., Dias Filho B.P., de Abreu Filho B.A. (2017). Bioactivity of Essential Oils in the Control of Alternaria Alternata in Dragon Fruit (Hylocereus Undatus Haw.). Ind. Crop. Prod..

[53] Jadhav S., Shah R., Bhave M., Palombo E.A. (2013). Inhibitory Activity of Yarrow Essential Oil on Listeria Planktonic Cells and Biofilms. Food Control..

[54] Fierascu I., Ungureanu C., Avramescu S.M., Fierascu R.C., Ortan A., Soare L.C., Paunescu A. (2015). In Vitro Antioxidant and Antifungal Properties of *Achillea Millefolium* L. Rom. Biotechnol. Lett..

[55] Khan F.A., Khan N.M., Ahmad S., Nasruddin, Aziz R., Ullah I., Almehmadi M., Allahyani M., Alsaiari A.A., Aljuaid A. (2022). Phytochemical Profiling, Antioxidant, Antimicrobial and Cholinesterase Inhibitory Effects of Essential Oils Isolated from the Leaves of Artemisia Scoparia and Artemisia Absinthium. Pharmaceuticals.

[56] Polatoğlu K., Demirci B., Demirci F., Gören N., Başer K.H.C. (2012). Biological Activity and Essential Oil Composition of Two New Tanacetum Chiliophyllum (Fisch. & Mey.) Schultz Bip. Var. Chiliophyllum Chemotypes from Turkey. Ind. Crop. Prod..

[57] Mssillou I., Agour A., Allali A., Saghrouchni H., Bourhia M., El Moussaoui A., Salamatullah A.M., Alzahrani A., Aboul-Soud M.A.M., Giesy J.P. (2022). Antioxidant, Antimicrobial, and Insecticidal Properties of a Chemically Characterized Essential Oil from the Leaves of *Dittrichia Viscosa* L. Molecules.

[58] Gilmore B.F., Flynn P.B., O’Brien S., Hickok N., Freeman T., Bourke P. (2018). Cold Plasmas for Biofilm Control: Opportunities and Challenges. Trends Biotechnol..

[59] Arce Miranda J.E., Sotomayor C.E., Albesa I., Paraje M.G. (2011). Oxidative and Nitrosative Stress in Staphylococcus Aureus Biofilm. FEMS Microbiol. Lett..

[60] Barraud N., Hassett D.J., Hwang S.-H., Rice S.A., Kjelleberg S., Webb J.S. (2006). Involvement of Nitric Oxide in Biofilm Dispersal of Pseudomonas Aeruginosa. J. Bacteriol..

[61] Fang F.C., Vázquez-Torres A. (2019). Reactive Nitrogen Species in Host–Bacterial Interactions. Curr. Opin. Immunol..

[62] Arasimowicz-Jelonek M., Floryszak-Wieczorek J. (2016). Nitric Oxide in the Offensive Strategy of Fungal and Oomycete Plant Pathogens. Front. Plant Sci..

[63] Mayrs E.B.C. (2017). British Pharmacopoeia Appendix 9.

[64] Adams R.P. (2007). Identification of Essential Oil Components by Gas Chromatography/Mass Spectrometry.

[65] Ahmed A.F., Attia F.A.K., Liu Z., Li C., Wei J., Kang W. (2019). Antioxidant Activity and Total Phenolic Content of Essential Oils and Extracts of Sweet Basil (*Ocimum basilicum* L.) Plants. Food Sci. Hum. Wellness.

[66] Thaipong K., Boonprakob U., Crosby K., Cisneros-Zevallos L., Hawkins Byrne D. (2006). Comparison of ABTS, DPPH, FRAP, and ORAC Assays for Estimating Antioxidant Activity from Guava Fruit Extracts. J. Food Compos. Anal..

[67] Olszowy M., Dawidowicz A.L. (2016). Essential Oils as Antioxidants: Their Evaluation by DPPH, ABTS, FRAP, CUPRAC, and β-Carotene Bleaching Methods. Mon. Für Chem.-Chem. Mon..

[68] Bagla V.P., McGaw L.J., Elgorashi E.E., Eloff J.N. (2014). Antimicrobial Activity, Toxicity and Selectivity Index of Two Biflavonoids and a Flavone Isolated from Podocarpus Henkelii (Podocarpaceae) Leaves. BMC Complement. Altern. Med..

[69] Asong J.A., Amoo S.O., McGaw L.J., Nkadimeng S.M., Aremu A.O., Otang-Mbeng W. (2019). Antimicrobial Activity, Antioxidant Potential, Cytotoxicity and Phytochemical Profiling of Four Plants Locally Used against Skin Diseases. Plants.

